# Releasing Operating Room Nursing Time to Care through the Reduction of Surgical Case Preparation Time: A Lean Six Sigma Pilot Study

**DOI:** 10.3390/ijerph182212098

**Published:** 2021-11-18

**Authors:** Patricia Egan, Anthony Pierce, Audrey Flynn, Sean Paul Teeling, Marie Ward, Martin McNamara

**Affiliations:** 1 Beacon Hospital Beacon Court, Bracken Rd, Sandyford Business Park, Sandyford, Dublin 18, D18 AK68 Dublin, Ireland; anthony.pierce@beaconhospital.ie (A.P.); Audrey.flynn@beaconhospital.ie (A.F.); 2 UCD Centre for Interdisciplinary Research, Education & Innovation in Health Systems, School of Nursing, Midwifery & Health Systems UCD Health Sciences Centre, D04 V1W8 Dublin, Ireland; sean.p.teeling@ucd.ie (S.P.T.); martin.mcnamara@ucd.ie (M.M.); 3 Centre for Innovative Human Systems, School of Psychology, Trinity College, The University of Dublin, D04 V1W8 Dublin, Ireland; Marie.Ward@tcd.ie

**Keywords:** preference card, surgical materials, Lean Six Sigma (LSS), custom pack, touchpoints, operating room (OR)

## Abstract

Healthcare systems internationally are working under increasing demand to use finite resources with greater efficiency. The drive for efficiency utilises process improvement methodologies such as Lean Six Sigma. This study outlines a pilot Lean Six Sigma intervention designed to release nursing time to care within a peri-operative environment; this was achieved by collaborating with stakeholders to redesign the process for laparoscopic hernia surgical case preparation (set up) material. Across 128 laparoscopic hernia surgical cases, the pilot resulted in a 55% decrease in overall nursing time spent in gathering and preparing materials for laparoscopic hernia surgical cases, with a corresponding reduction in packaging waste. The major impact of releasing nursing time to care within busy Operating Room environments enabled nurses to focus on continuing to deliver high-quality care to their patients and reduce pressure expressed by the Operating Room nurses. The results have led to an ongoing review of other surgical procedures preparation to further release nursing time and will be of interest to perioperative teams internationally.

## 1. Introduction

An operating room (OR) is the unit of a hospital where surgical procedures are performed [1]. The hospital OR team is a highly-skilled multidisciplinary team of healthcare staff working concurrently across several OR suites. The environment within an OR is considered clean; it has restricted access to OR personnel who make use of impermeable surgical drapes, hospital-laundered surgical scrubs, masks, and modern surgical gowns—all elements of infection control in the operating room [2]. Universally, hospital surgical services in the OR are central to the quality and safe delivery of healthcare and, as they are a hospital’s largest cost and revenue centre [3], have a major impact on the performance of the hospital as a whole. OR managers aspire to increase departmental efficiency by reducing preparation time for surgical cases and turnaround time (time between surgical cases) of individual suites [4]. The role of the OR nurse may include anesthesia, surgical case assistance, or attending to the Post-Acute Care Unit (PACU). The surgical or OR nurse works as part of the surgical team as a surgical case assistant, a patient advocate, and a coordinator in the preparation and use of surgical tools and materials for surgery, all the time maintaining a clear focus on patient care before, during and after surgery [5]. This study site is a private hospital in Dublin, Ireland with a high surgical output through its eight ORs and over 17,000 scheduled surgeries per year. There are over 200 surgeons working in different specialties, including orthopedics, urology, and general surgery. The hospital has been committed to adopting Lean Six Sigma (LSS) methodology for process improvement since 2017 through an onsite training Academy partnered with University College Dublin (UCD). LSS is a combination of two process improvement methodologies developed in industry, Lean and Six Sigma [6]. LSS in healthcare is the philosophy of improving the flow of patients, information, or goods whilst eliminating waste in the process [7], which can be achieved by ‘understanding current processes, identifying the areas for improvement and implementing necessary change’ [8] (p. 679). The use of LSS in the OR environment has been shown to increase the safety and reliability of care, improve team performance and staff wellbeing, and add value and improve efficiency [9]. Additionally, LSS has been shown to be synergistic with person-centred approaches that have a focus on both patient and staff experiences of care [10]. Person-centred approaches to care have been shown to lead to increased satisfaction among healthcare staff about the quality of the care they deliver [11].

In 2018 a cross-functional team in the hospital worked on a project streamlining OR storage by reducing the number of surgical materials by 20% and re-organising the storage area layout to reduce the time spent by OR nurses looking for essential surgical materials, save costs in wasted stock and release nursing time to care [12]. As part of the organisation’s ongoing commitment to quality improvement, in 2020 a multidisciplinary team of employees began work on a further LSS project to optimise the process further. The project focused on the reduction of OR nursing time spent collecting materials used in surgical lists. Each specific case for each surgeon (n = 200) has its materials and equipment requirements outlined on an associated preference card. The individual surgeon advises what is listed on the procedure preference card, and the individual surgeon’s card is updated on an ad hoc basis. The preference cards are a mixture of typed and handwritten, i.e., there is no central electronic database. An OR nurse selects the correct preference card for the surgery and then collects the materials from the storage areas in preparation for the following day’s surgical list. This is performed at the later end of the nurses’ 12-h shift, to allow for any changes to the order of the operative list that may occur in planning during the day. Given the number of surgeons and diversity in surgeries, there is a large inventory of these preference cards. Additionally, the hospital has seen an increase of 25% in the number of surgeries performed since 2015.

Throughout the literature, LSS deployment in healthcare is highly dependent on extensive stakeholder engagement through repeated Voice of the Customer (VOC) feedback [13,14,15] and this includes the busy environment of the OR [16,17]. Voice of the Customer (VOC) is a term that describes a customer’s feedback about their experiences with and expectations from services, with a focus on customer needs, expectations, understandings, and process improvement [10]. An initial VOC from OR nursing staff about the process for the collection of materials for surgery indicated that they felt that some of their time could be released if they spent less time on this process of gathering and preparing materials for individual surgical cases. This gathering and preparing process for materials was classified as containing periods of Non-Value Add (NVA) time, activities that involve work that consumes resources but does not add value to the product or service [17]. If excessively present, NVA can result in customer dissatisfaction [18], in this case with the OR nursing team. 

The hypothesis of this study was that if the NVA time the OR nurses referred to in the VOC could be quantified, the process could be redesigned to reduce it. From the broad range of surgical services to choose for a pilot study, laparoscopic hernia surgeries were determined as a suitable focus. This was firstly due to the frequency of these surgeries in the hospital, which has increased 129% in 5 years, even seeing an increase during the Covid-19 pandemic year (March 2020 to present) and gave a good sample size for the study. Further, five different surgeons were performing these surgeries and each surgeon had a unique preference card that lists the preferred surgical materials for their case, giving a diverse sample of surgeons, but each with their specific preferences. This was deemed a suitable starting point for a wider, long-term strategy of the hospital to optimise the process involving OR nursing time assigned to prepare for surgeries and to maximise the nursing time available to patient care.

## 2. Methods

The team used the LSS framework of Define, Measure, Analyse, Improve, and Control (DMAIC), a data-driven quality improvement strategy used to improve processes [19]; each word represents a continuum of the five phases that make up the process, and in this paper, we structure our methods within each of the phases. The DMAIC framework was used to structure the engagement with stakeholders, data collection and analysis and to facilitate the development of a plan for and implementation of improvements. Improvements were co-designed with stakeholders who were working in, on, or with the current process for the collection of materials for surgery, predominantly nursing and procurement teams, and we used LSS tools appropriate to the improvement (Table 1).

Jones [20] suggests that what distinguishes LSS from other process improvement methodologies is its focus on developing the capabilities of teams (doctors, nurses, and administrative and support staff) to manage and continuously improve their work. When an organisation begins to adopt LSS, individual and team-based learning is the focus, not just in the classroom, but in the practice area. Jones [20] further argues that it is through this application of Lean that healthcare staff can remove unwanted NVA activities, giving them more time to spend with patients. The multidisciplinary project team included a Physiotherapist, Radiology Manager, Project Coordinator, OR procurement team member, and OR coordinator, a nurse. All team members were involved at all stages with the physiotherapist and radiology manager and project manager in performing and analyzing a series of observational studies. The DMAIC phases are now used to outline the methods used within this improvement project.

### 2.1. Define

**Figure 1:**
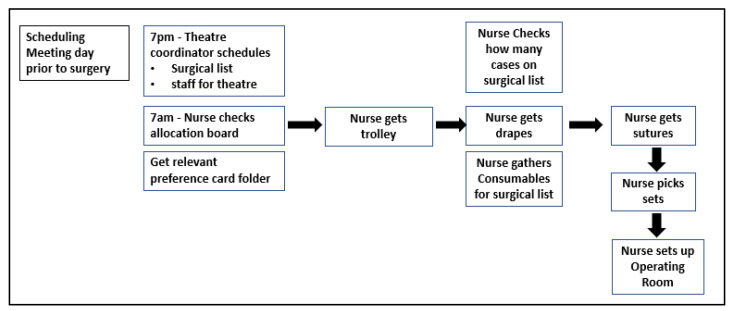
Original ‘as we think it is’ Process Map.

There was no baseline data available for the preparation time for laparoscopic hernia cases, as preparation of materials for surgery was not routinely timed. This was therefore gathered through an initial observational study, in LSS terms, a ‘Gemba’, the aim of which was to go to the actual place of work, observe the process steps in real-time, and record the observations and any other relevant information [21]. Gemba walks were carried out by project team members unfamiliar with OR processes to allow for a fresh perspective and to ask critical questions about the OR nurses’ experience of the process. This form of observational study had previously been carried out in the OR but had focused solely on the process steps in the storerooms. These Gemba facilitated the development of an ‘as we think it is’ map for the original process (Figure 1).

The project stakeholders were initially identified by looking at the original ‘as we think it is’ process map for material collection for surgery, and those staff members who were working in, on, or with this process and that could either influence or be impacted by the process. These stakeholders included five General Surgery consultants, the hospital’s Corporate team, comprising of the Chief Financial Officer, the Director of Clinical Hospital Operations, Head of Surgical Services, and their team incorporating the OR manager and OR Clinical Nurse manager, along with the procurement manager. The VOC sessions used a combination of questionnaires with the nurses most closely involved in the process and follow-up interviews with them and the other stakeholders. The OR nurses (n = 35) received questionnaires with a series of closed questions, to gain quantitative insight, and open questions to understand the current process for gathering and preparing surgical materials for OR lists and their experiences of, and feelings about, the task. The other stakeholders (n = 11) with management responsibilities, although not directly involved in the materials preparation process, were chosen for an interview to assess their perception of the process and its impact on staff and patients. This approach enabled us to gather a system view of the problem from the combined perspective of individuals. Data was gathered over three weeks. The project team took a person-centered approach throughout all interactions with stakeholders. Putting the person in the center as a priority for proper care and good and efficient healthcare services [21], person-centred approaches are highly effective in LSS interventions [10,22,23,24] and the project team was collaborative, inclusive, and encouraged stakeholder participation at all stages of the study. 

**Figure 2:**
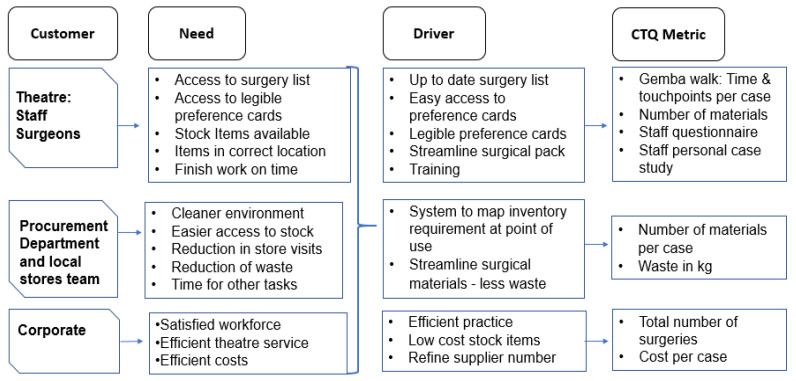
Critical to Quality Tree.

The engagement with a wider group of stakeholders aided the development of a Critical to Quality tree (CTQ) (Figure 2). A CTQ helps businesses to define and meet customer needs by capturing the key measurable elements the customer needs, the characteristics by which the quality of service or process is judged (driver), and the requirements to meet those standards to satisfy the service user [25]. In this case, the CTQ enabled the project team to develop metrics translated from our captured VOC (Table 2) by mapping their specific identified needs, the drivers required to attain these, and what data was required to capture that, illustrated where the drivers delivered/did not deliver on those needs.

### 2.2. Measure

The Gemba revealed that the established process for laparoscopic hernia preparation (Figure 1) began with obtaining the surgical list for the specific surgeon; then the preference card folder for the surgeon was sourced; this folder holds each of the preference cards for the types of surgery that specific surgeon performs. Many of the preference cards are handwritten. The OR nurse then collected a trolley from a theatre location, opened a plastic bag to hold the materials for each case, and then gathered surgical materials required as they appeared on the preference card. The OR Storeroom is a narrow awkward shape measuring 40 square meters. Observation data indicated that the materials were gathered in a plastic bag, held on a trolley, wheeled to the theatre, and stored there for use in the operative list the next morning. The surgical materials associated with a particular case were then opened directly before the surgery in the OR. Each item was opened in a manner that maintains the sterile integrity of the material. This was seen to be time-consuming and produced packaging waste in a busy OR. The preparation time was 4 min 36 s per case across five Gemba, which incorporated 24 cases. Working from this data the project team aimed to reduce the nurse’s preparation time by 50%, as the initial VOC from OR nurses working with the processes felt that this was ‘lengthening an already long shift (12 h)’, reported feeling ‘drained and annoyed’, and unable to attend to the task due to ‘interruptions if there are ongoing cases’. 

**Figure 3:**
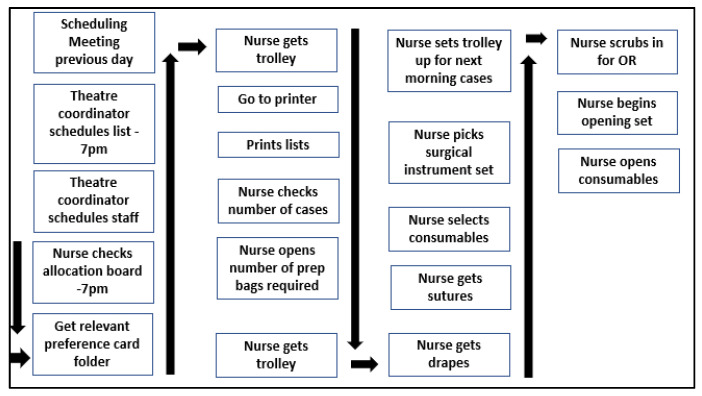
Validated ‘as is’ current state process following the Gemba.

Gemba walks were performed over 3 months to establish the reality of the situation by verifying the qualitative information gleaned from questionnaires and interviews. The Gemba walks were carried out by the project team who each followed the process of the nurse selecting surgical materials for the lists. A touchpoint was defined as any physical interaction of the OR nurse with an item involved in the process [26]. Each touchpoint and duration of the steps was recorded during the Gemba walk. This data refined the initial process map (Figure 1), giving a validated ‘as is’ overview of the process, or a ‘current state’ map (Figure 3). The data collected included time taken for the OR nurse to get the case list and preference card folder; time it took to gather the surgical materials stated on the preference card for each case to take place on that surgeon’s list; the total number of touchpoints; in next day surgery, the time it took to open all the surgical materials for the surgery; and the dry waste generated from the packaging of the consumables. 

The data collected from the nurse questionnaires (Table 2) gave the project team both an understanding of the NVA for the OR nursing team and the potential impact this had on their satisfaction with the current process. It was evident that there was a wide variation in the process and that it was time-consuming for staff already tired at the latter end of a busy work shift. Kinsman et al. [27], discuss how staff satisfaction with the process and impact of LSS deployment are considered an important outcome for evaluation. 

### 2.3. Analysis

The data gathered in the Gemba walks aided an understanding of the impact of touchpoints, the time spent at the individual steps, and the waste produced. The project team took the perspective of a nurse selecting materials for:

1. A laparoscopic hernia surgical list
2. All laparoscopic hernia cases in the year.

This gave the project team an understanding of the NVA in the current process and areas of potential room for improvement. The project team utilised the TIMWOODS tool to further refine NVA identified in the Gemba and process maps. TIMWOODS is an anacronym, wherein each letter stands for one of eight potential wastes: transport, inventory, motion, waiting for time, over-processing, overproduction, defects, and skills [28]. Concepts are interrelated and reducing or eliminating one of them can and will affect the others. The VOC had shown the project team that staff felt overburdened and pressured by this task, delaying getting home on time, being taken away from this task to complete other more patient-centric work, and therefore not completing the material preparation for surgical lists on time. Themes emerged across all eight wastes providing the opportunity to improve (Table 3).

**Figure 4:**
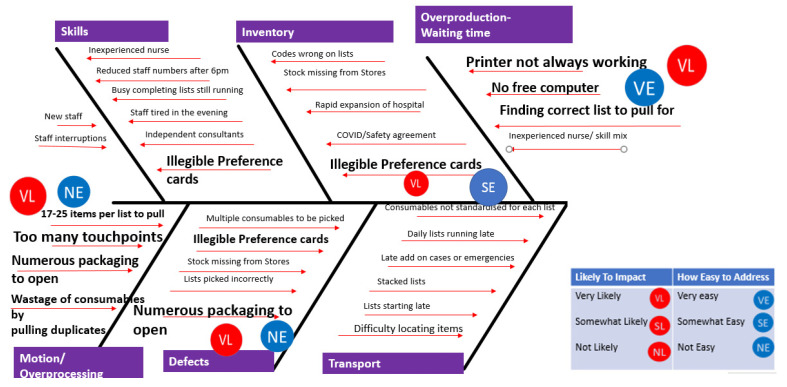
Fishbone (Ishikawa) diagram.

Ishikawa (or fishbone) was used to further delve into the identified themes (Figure 4). Ishikawa helps detect different types of variation within a process [29]. The deep dive presented the project team with two possible ‘easy to effect, high impact solutions in the form of (a) having the surgical list pre-printed for the OR nurse before s/he started the process; (b) having typed, legible preference cards. The results of the TIMWOODS and Ishikawa analyses were then reviewed and discussed with the stakeholders.

### 2.4. Improve

The stakeholders and project team agreed on the need to reduce the OR nurses’ time for gathering the surgical materials for laparoscopic hernia cases. Walking through the process, the project team brainstormed potential creative solutions with the key stakeholders by using the findings of the Gemba to inform and complete their TIMWOODS and Ishikawa. Themes identified as possible solutions included finding common consumables, creating legible preference cards, reducing touchpoints, and making surgery lists easily available. The stakeholders most directly impacted by the proposed improvements were the General Surgery consultants and OR nurses. Hearing the nurses’ needs was important to the project team and became a common goal, as they had expressed that the current process for surgical material collection was a time-consuming task, which added considerably to an already long day (12-h shift). 

**Figure 5:**
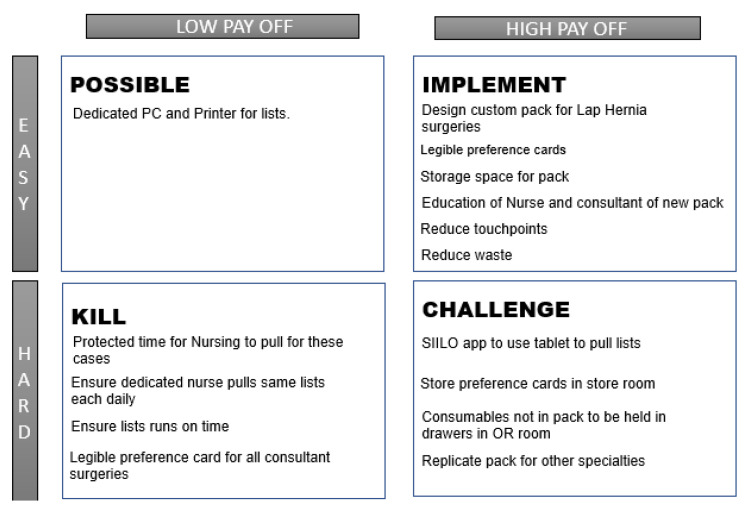
PICK Chart.

The project team and stakeholders used a PICK chart to visually identify and organise ideas into categories of low impact—easy to implement, high impact—easy to implement, high impact—hard to implement, low impact—hard to implement (Figure 5). PICK—Possible, Implement, Challenge, Kill—was used to prioritise the potential improvements to give the biggest payoff, allowing for the nurses to feel heard [30]. Several solutions were presented, discussed, and recorded using a practicality tool [31] which ranked potential solutions in terms of:

- Way out there,
- Quite impractical,
- Might be workable,
- Close to workable, and
- Could implement today.

The solution which ranked as close to being workable and which could have a positive impact on reducing waste in its varied forms—time, packaging, motion—was a customised surgical pack. A custom pack combines the surgical materials into a single sterile package that is used for specific types of surgery. The 5 S tool [32]—sort, set in order, shine, standardise, and sustain—was then used to develop the concept for the management of the implementation of the custom pack in the OR. The stakeholders involved in this were the surgeons, the OR nurse, and Procurement. There were 27 common surgical materials identified across the five surgeon preference cards for Laparoscopic hernia surgeries and it was agreed to adopt a common preference card to suit all laparoscopic hernia cases.

There were 54 touchpoints associated with these 27 common surgical materials. A standardised custom pack would reduce these touchpoints dramatically to two key touchpoints and then several other ancillary touchpoints. A prototype custom pack was co-designed with the OR nurses and surgeons, and discussed with and ordered with an existing hospital supplier. This process took 2 months to be completed as it required validating stakeholder needs and arriving at a custom pack prototype that suited all end users. When the prototype was received the Gemba of the process for laparoscopic hernia case preparation was repeated. 

## 3. Results

Data were recorded over three months between November 2020–January 2021. Gemba walks observing 24 laparoscopic hernia cases identified quantitative data for process time and touchpoints. Weight of waste and waste in motion (Figure 5) were also recorded during this time. Qualitative data was recorded via Voice of Customer surveys with each stakeholder group and post-intervention OR nurse interviews were conducted. Following the introduction of the codesigned prototype custom pack, the second set of measurements occurred over 1 month in April 2021. 

**Figure 6:**
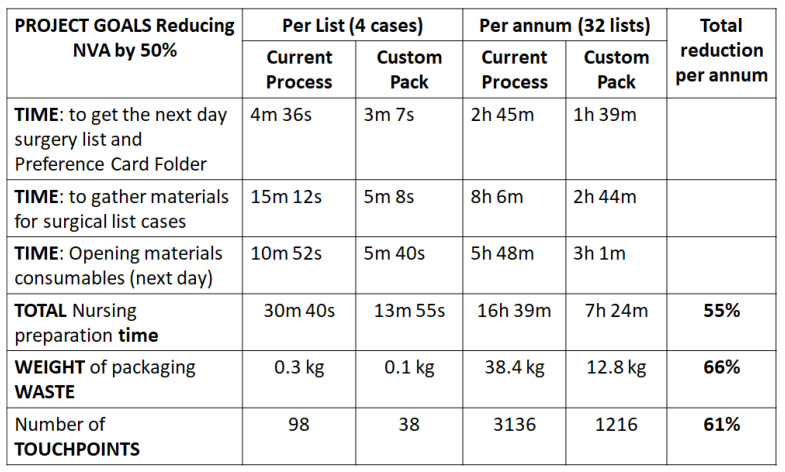
Comparison results from pre- and post-Custom packs.

**Figure 7:**
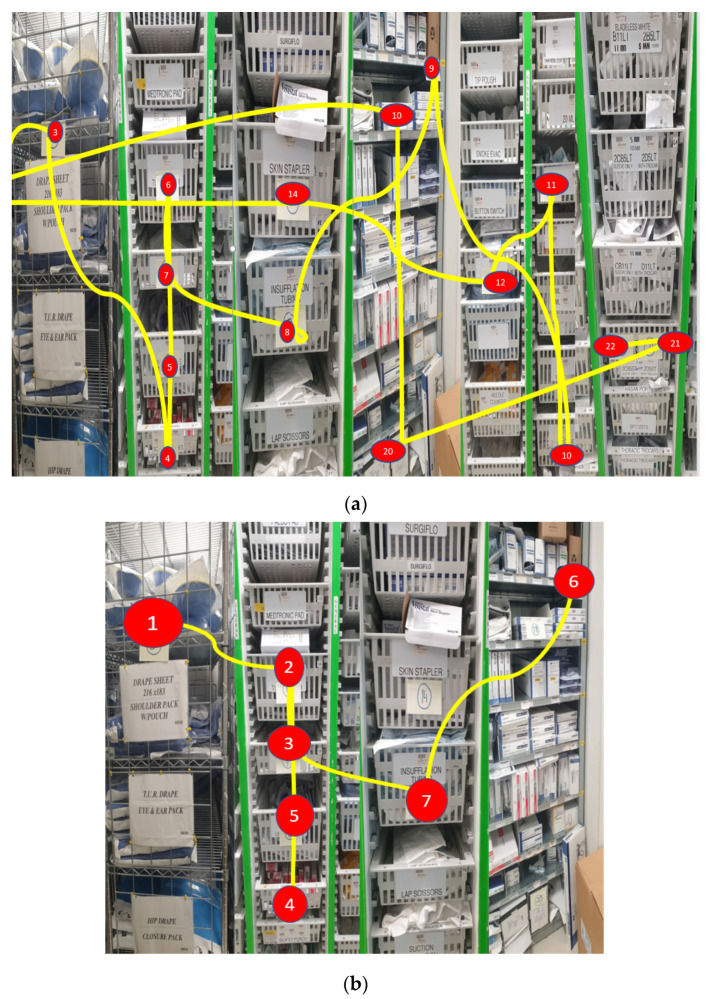
Photos demonstrating the number of touchpoints (**a**) pre- and (**b**) post-custom pack.

The identified reduction of NVA, potentially achievable with the introduction of the prototype custom pack in comparison to the current (original) process, is detailed in Figure 6. This was verified using Gemba walks. The results are outlined below:

- The time to locate a surgeon’s surgical list and associated preference card reduced by 32% from 4 min 36 s to 3 min 7 s.
- Time collecting materials as listed on preference card per average surgical list of 4 cases reduced by 10 min;
- Time spent opening consumables almost halved, saving just under 5 min of opening individual materials’ packaging for each surgical case;
- The overall OR nurse time saved in the process when using the custom pack was 16 min 45 s or 55%;
- Number of storage locations (touchpoints) the OR nurse had to access to collect materials reduced by 66% from a total of 98 to 38;
- Weight of waste packaging fell to one third from the original 0.3 kg to 0.1 kg per case;
- Custom pack decreased the number of touchpoints (Figure 7), which in turn reduced the repetitive mechanical motion associated with reaching for materials in the storeroom.

The current plan is to incrementally introduce the custom pack concept across all eight OR suites. Given the evidenced 26-min time saving per list, we extrapolate that running two lists per day (Monday to Friday) per OR suite will yield an overall annual time saving of 1800 h of OR nursing time.

The VOC of the OR nurses was once again acquired using a questionnaire (Table 4). There was a response rate of 73% (11 responded out of a possible 15). Positive results were recorded when the prototype custom pack was used.

### Control

Following the trial of the custom pack and having identified the benefits of such a pack the voice of the customer was revisited. Once again, this engaged the stakeholders to record their opinions on this proposed solution and whether they would be comfortable to proceed with this change in the process. This resulted in the surgeons and OR nurses feeling empowered in designing a plan to implement a custom pack for laparoscopic hernia surgeries (Table 5 Control Plan). It is important to note that speaking to the Consultant Surgeons (n = 5), that they were “happy”. One consultant quoted that ‘a complete pack is a great idea, I am happy’.

## 4. Discussion

The original goal for this study was to release nurse time to care. Following VOC sessions with key stakeholders, we hypothesised that this could be achieved by reducing NVA in the established process for gathering and preparing materials for next-day surgical lists. A goal of a 50% reduction in nurse time spent on certain steps in the process was expected. This was to improve the experience of the OR nurse and release the nurse back to what they do best, i.e., clinical work, being with their patients. The extrapolated data in Table 4 demonstrates the baseline data compared to a potential reduction in time, touchpoints, and NVA can give savings greater than 55% across those three categories. Introducing a custom pack offered other tangible positive outcomes to the OR nurse who leads this process. Custom packs also reduced the packaging waste associated with individual consumables, introducing further cost savings.

The project team spoke of reducing pressure on staff within the OR, in particular, OR nurses as represented in our VOC (Table 4). Gustavsson et al. [33] suggest that whilst the patient’s view is paramount in determining healthcare quality, this is better served by also including the views of the healthcare professionals caring for patients who work within the system and know the culture. Jones [20] suggests that what distinguishes Lean from other process improvement methodologies is its focus on developing the capabilities of healthcare teams (doctors, nurses, and administrative and support staff) to manage and continuously improve their work. The hospital has an academy that promotes and embeds LSS methodology throughout the organisation by providing introductory courses (white belt) in LSS to all staff, as well as supporting 12 to 16 professional certificate (green belt) courses places per year. Graduate Diploma and MSc level (black) belt training has also been introduced through a post-graduate course with UCD. The Academy structures and systems supported the project team in their improvement. Lean Six Sigma utilises a bottom-up, top-down approach which means that healthcare staff is enabled to examine their work processes, collect and analyse their own data and implement their own solutions [33,34].

According to Castle and Harvey [35], this allows for rapid root cause analysis and genuine staff involvement, with staff leading on projects to improve patient outcomes. This bottom-up, top-down approach to LSS in the study site facilitated engagement with the OR team, and in particular the OR nursing team. O’Neill et al. [36] suggest that nursing professionals are well placed as decision-makers to identify and meet patient needs and argue that a bottom-up approach to process improvement works well. Johnston [37] suggests that nursing is a profession ideally suited to Lean deployment as its members have extensive experience of working in and leading interdisciplinary teams, are patient-focused, and can view the healthcare system from the patient’s viewpoint. Jones and Woodhead [38] reiterate that Lean skills are learned through daily practice and not just from classroom training in Lean tools or occasional workshops. Joosten et al. [39] believe that managers must focus not only on process improvement but also on developing their staff through support, respect, and education, as ultimately it is the staff who will implement any change process.

The project team predominantly looked at improving efficiency by working in person-centered collaborative, inclusive, and participatory ways with colleagues in the OR to achieve this efficiency whilst reducing pressure on the busy OR nursing team. The project team further emphasised coming together as a healthcare team and creatively designing solutions that were important to all stakeholders, such as improving staff wellbeing through releasing time to care for busy healthcare staff under constant time pressure. Importantly, participants expressed the view that this outcome was an important factor in their receptivity to LSS, through the process of engaging healthcare staff in defining the problem and involving them in designing the solution, which has the potential to transform individual departments right through the whole organisation. Transformation means change, which could mean going from a place of inertia or switching direction and momentum. Change evokes both analytical and emotional responses [39]. Change is often resisted due to misunderstanding, parochial ‘self-interest’, differing expectations, or fear. The project team was cognisant of this during stakeholder engagement and applied active listening to focus on problems important to staff in the OR. The key focus of this project was to release OR nursing time to care for patients. Research investigating this problem of OR time insufficiencies has been widely investigated previously [40]. Similar to our project, Koyle et al. [41] used Lean observational methodology to analyse OR nurse cycle time in surgical tray preparation. An improvement of 6 min (pre-intervention time 11 min vs. post intervention time 5 min) per tray was documented following the introduction of a standardised surgical pack [41]. This percentage improvement of 55% corresponds with our results exactly (Table 4) with a 3.5-min improvement post introduction of the custom pack (pre-intervention time 7 min vs. post-intervention time 3.5 min). Although Koyle et al. [41] examined the impact of a standardised pack on OR nurses’ time in a different speciality (paediatric cases), the similarities in outcome provide support for our Lean approach.

A key factor in reducing preparation time for OR nurses was the need to decrease the number of consumables selected across five highly experienced autonomous surgeons. To achieve this, each surgeon’s preference card was reviewed for consumable similarities with their colleagues, and a cost analysis was completed before a single consumable pack was provided for their consideration. This resulted in a reduction of 21 items (Table 4) for OR nurses to select for surgery. Avansino et al. [42] followed a similar approach of preference card review with follow-up stakeholder engagement. A total of nine items were reduced from the list with the introduction of a single preference card [42]. Although this study examined all instruments rather than just consumables, the project was promoted by the Division of General Surgery [42]. The initial recognition at the surgeon level of the need to decrease instrument number is considered key for the positive outcome and success of a project aiming to reduce preference card items [43]. Although, this was not the case in this study, with OR nurses first to consider alternate options, surgeon engagement and the drive for change were likely to have been assisted by the opportunity to peer review their colleague’s preference card and costs [44].

Limitations of this study are that it was on a single surgical service within the hospital. However, we did select a specialty with five surgeons each utilising a different consumable list on their preference card. Eight operating theatres serve a multitude of other surgical services. The potential to introduce the custom pack whilst maintaining the same level of care to patients is obvious, with this study reflecting a 55% reduction in total nursing time (at multiple steps) in the process. There have already been initial discussions to introduce a similar custom pack to cardiology and ophthalmology surgery within the hospital. This template can be replicated in other hospitals but does require stakeholder engagement. Another potential limitation of this study was the lack of direct patient engagement in reviewing the process. Our process map did not include direct patient contact due to the nature of analysing theatre store activity. However, we would argue that the breadth of multi-disciplinary team members who responded to our surveys reflected the patient’s journey through all stages of their surgery. The range of MDT involvement is viewed as a real strength of this project. The combined skills and disparate backgrounds of the members of the project team gave an interesting dynamic to the team who worked together over 10 months to complete the work. This project has the potential to be transformational to the organisation. From a system thinking perspective, it allowed reviewing patterns of systemic relationships and associated processes.

## 5. Conclusions

Change is a complex process, and to be successful buy-in from those directly impacted when delivering the service, OR nurses and surgeons are required. Stakeholder engagement is identified in LSS as one of the most important tools to ensure the success of a project. From the outset the project team was concerned with the amount of time nurses spent on the preparation of materials for surgery, thus taking them away from patient care. This project has successfully evaluated and quantified NVA in nursing time, packaging, and costs. The project team identified potential ways to reduce this NVA seeing a total reduction in OR nurse preparation time of 55% post-implementation of the custom pack. This results in more efficient use of nursing time to focus on clinical duties resulting in more OR nurse time to focus on the patient experience. It presents a formula for the successful application of LSS methodology to this problem and has proven a template for other service streams in the operating room.

## Figures and Tables

**Table 1:** LSS Tools.

Title of Improvement Tool	LSS DMAIC–Define, Measure, Analyse, Improve, Control Is a Data-Driven Quality Strategy Used to Improve Processes
Stakeholder engagement	Consultation with stakeholders to understand the problem, expectations of stakeholders and derive potential solutions
VOC	A customer’s feedback about their experiences with and expectations from services
NVA	Time and/or activities that involve work that consumes resources, but does not add value to the product or service
Gemba	Observation of the process steps in real-time; observations and other relevant information is recorded
CTQ	Helps businesses to define and meet customer needs by capturing the key measurable elements the customer needs, the characteristics of which the quality of service or process is judged (driver) and the requirements to meet those standards to satisfy the service user
Process map	Map showing the steps of the original process both ‘as we thought it’ and ‘as is’ following validation
TIMWOODS	A useful tool wherein each letter stands for one of eight potential wastes: transport, inventory, motion, waiting time, over-processing, overproduction, defects and skills.
Ishikawa/fishbone	Through brainstorming this helps detect different types of variation within a process
5S	Sort, Set in order, Shine, Standardise, and Sustain–used to develop the concept for the management of the implementation of a potential solution
PICK Chart	Possible, Implement, Challenge, Killed chart a visual tool to prioritise the potential improvements to give the biggest pay off
Control Plan	Defines for each process step an associated metric (measure) and status (expected timeline or responsible person)

**Table 2:** Quantitative data—VOC questionnaires.

Questions Asked-OR Nurses (Staff)	Responses (N = 14)
Over the last fortnight have you had to collect materials for surgical cases?	Yes = 14 No = 0
How long does it take you to pull for cases for the following day?	15 min to 1 h
Do you ever get interrupted when performing this task?	Yes = 14 No = 0
Have you ever stayed late to perform this task?	Yes = 14 No = 0
How long have you stayed late? (range of responses)	15 to 45 min
Over the last fortnight how many times have you had to pull for cases?	Every day on shift

**Table 3:** TIMWOODS demonstrates identified themes of NVA.

	NVA	Impact	Identified	Solution
T	Transport	Moving items	Excessive Time spent picking items	Find common consumables across preference cards
I	Inventory	Items unavailable	Preference card in different formats and difficult to locate	Create a standard electronic format
M	Motion	Excessive movement in small workspace	Excessive Touchpoints–ergonomics in selecting 17–27 items per pack	Find common consumables across preference cards
W	Waiting time	Time spent searching for preference card	Staff searching for the correct Preference card	Pre-printed preference card/assigned and ready to collect from desk
O	Over-processing	Doing more than necessary	Excessive touchpoints–consistency in items required across surgeries	Find common consumables across preference cards
O	Over-production	Excess items Packaging waste	Staff picking numerous common consumables Reduce packaging waste	Find common consumables across preference cards
D	Defects	Mistakes or errors requiring rework	Potential to omit items that are not in the stock room	Pre-printed preference card and common consumables
S	Skills	Not using worker for their abilities	Excessive Time spent on picking items	Pre-printed preference card and common consumables

**Table 4:** VOC of OR Nurses post-5S custom pack solution.

	Y	N	% Agreement
Do you think this proposed pack will save you time?	11	0	100%
Do you think this proposed pack will be easy to use?	11	0	100%
Is pulling a list physically demanding? Would this pack make it physically easier?	11	0	100%
Do you think a customised pack would be useful in other surgical specialities?	10	1	91%
Do you think a customised pack will reduce the number of missing items when packing?	10	1	91%

**Table 5:** Control plan showing the process step associated metric and status at the time of paper submission.

Process Step	Critical Improvement Metric	Status
Reduce paper waste	Custom pack removes 21 items	Complete
Common typed preference card	Reduces time to pull for lists following day	Complete
Prototype Custom pack ordered for lap hernia procedures	Reduces touchpoints Eliminates non-value add Reduces paper waste	Complete
Procurement at tender	Due Diligence and Quality control	Assigned OR nurse
Validate custom pack/Staff education	Quarterly monitoring of quality of custom pack	Assigned OR nurse

## Data Availability

The data presented in this work are available in the paper.
